# Epidemiological Evidence and Health Risks Associated With Agricultural Reuse of Partially Treated and Untreated Wastewater: A Review

**DOI:** 10.3389/fpubh.2018.00337

**Published:** 2018-12-06

**Authors:** Anthony A. Adegoke, Isaac D. Amoah, Thor A. Stenström, Matthew E. Verbyla, James R. Mihelcic

**Affiliations:** ^1^SARChI, Institute for Water and Wastewater Technology, Durban University of Technology, Durban, South Africa; ^2^Department of Microbiology, Faculty of Science, University of Uyo, Uyo, Nigeria; ^3^Department of Civil, Construction, and Environmental Engineering, San Diego State University, San Diego, CA, United States; ^4^Department of Civil & Environmental Engineering, University of South Florida, Tampa, FL, United States

**Keywords:** epidemiological evidence, health risk, epidemics, exposure, antibiotics, pathogens, wastewater reuse

## Abstract

The use of partially treated and untreated wastewater for irrigation is beneficial in agriculture but may be associated with human health risks. Reports from different locations globally have linked microbial outbreaks with agricultural reuse of wastewater. This article reviews the epidemiological evidence and health risks associated with this practice, aiming toward evidence-based conclusions. Exposure pathways that were addressed in this review included those relevant to agricultural workers and their families, consumers of crops, and residents close to areas irrigated with wastewater (partially treated or untreated). A meta-analysis gave an overall odds ratio of 1.65 (95% CI: 1.31, 2.06) for diarrheal disease and 5.49 (95% CI: 2.49, 12.10) for helminth infections for exposed agricultural workers and family members. The risks were higher among children and immunocompromised individuals than in immunocompetent adults. Predominantly skin and intestinal infections were prevalent among individuals infected mainly via occupational exposure and ingestion. Food-borne outbreaks as a result of crops (fruits and vegetables) irrigated with partially or untreated wastewater have been widely reported. Contamination of crops with enteric viruses, fecal coliforms, and bacterial pathogens, parasites including soil-transmitted helminthes (STHs), as well as occurrence of antibiotic residues and antibiotic resistance genes (ARGs) have also been evidenced. The antibiotic residues and ARGs may get internalized in crops along with pathogens and may select for antibiotic resistance, exert ecotoxicity, and lead to bioaccumulation in aquatic organisms with high risk quotient (RQ). Appropriate mitigation lies in adhering to existing guidelines such as the World Health Organization wastewater reuse guidelines and to Sanitation Safety Plans (SSPs). Additionally, improvement in hygiene practices will also provide measures against adverse health impacts.

## Introduction

The reuse of partially treated or untreated wastewater for irrigation has been linked to diarrheal and parasitic infections (1–4) as well as skin disorder and other systemic infections (5–8). An effective managing strategy of these health risks is challenging and depend on water quality (e.g., the type of wastewater: untreated, partially treated, or treated) as well as societal and behavioral factors which will vary between different settings (9–11). Health risks are not only dependent on exposure to microbial hazards (i.e., infectious pathogens) but also to chemicals or pharmaceuticals (12–14).

Populations exposed to wastewater are most often categorized into four main groups;

a) agricultural workers and their families;
b) crop merchants, handlers, and technical/operational staff;
c) consumers of farm produce, vegetables, meat, or milk products;
d) residents of areas irrigated with wastewater, including children, the elderly, and immunocompromised individuals, who are at the highest risks in particular (15).

The first two groups (a and b) would mainly be exposed occupationally, due to direct contact with the wastewater during application and handling. Consumers (group c) are directly exposed dependent on their diet. Residents (group d) of areas close to sites irrigated with wastewater can also be exposed through farm run-off, groundwater contamination and aerosols.

Wastewater reuse has environmental sustainability benefits when practiced safely (16, 17). Adverse health outcomes associated with its reuse have been documented, both directly through epidemiological studies (2, 5, 18–23), and indirectly using quantitative risk assessments (10, 24–31). Despite these reported health outcomes, the use of wastewater for irrigation is a widespread practice, partly due to its year-round availability and nutrient content (32). It will thereby contribute to the urban food basket and the overall improvement of the urban environment (33).

To protect public health and ensure that the full benefits associated with the reuse are achieved, regional, national, and international authorities have set guidelines for safe wastewater use in agriculture [e.g., 34]. However, it may be questioned if wastewater use in agriculture still causes adverse health effects, despite the existence of these guidelines for safe use. The purpose of this review is to summarize the evidence associated with the health impacts resulting from exposure to pathogens in partially or untreated wastewater used for agricultural purposes. Specifically, we report on the relevant exposure pathways and summarize epidemiological evidence of diarrheal, intestinal parasitic, and skin infections associated with direct exposure as well as indirect through produce from agriculture. The later refer to foodborne outbreaks from irrigated produce. We review the estimated health risks from partially treated and untreated wastewater irrigation using quantitative microbial risk assessment, and the risks associated with contaminants such as antibiotics and antibiotic resistance genes. Finally, we conclude by presenting approaches to mitigating the risks associated with the use in agriculture.

## Methods

### Search Criteria

This review was based on literature searches in Science, Science Direct, PubMed, and Google Scholars till January, 2018. The keywords and word strings used in reference to microbial quality of wastewater intended for reuse were “wastewater OR treated wastewater OR partially treated wastewater OR untreated wastewater AND wastewater reuse AND microbial quality.” The health outcomes due to the reuse of wastewater was determined using the search string; “wastewater reuse AND epidemiological evidence OR diarrheal infections OR intestinal parasite infections OR infections OR microbial risk assessment OR quantitative microbial risk assessment (QMRA).” The last search string used in this review was; “wastewater reuse AND antibiotic residues OR antibiotic resistant genes OR antibiotic resistant bacteria.” The search results were reviewed by four reviewers.

### Inclusions and Exclusion Criteria

Selections were made without restriction on the study location or year of publication, but only articles written in English language were accounted for. Articles that address direct epidemiological evidence, health risks, and those that estimated the health risks indirectly through QMRA were collated. Others are those that considered crop contamination associated with partially treated and untreated wastewater use and resulting in human infection or ecological impact. A number of studies quantifying antibiotics and antibiotic resistance genes predicated by the wastewater reuse were also included due their associated risks.

### Extraction of Data

We specifically extracted and where appropriate tabulated information within the following areas:

a. Epidemiological evidence
b. Human health risks
c. Exposure pathways
d. Risk awareness
e. Ecotoxicity
f. Risk quotients
g. Risk mitigation

Tables and illustrative figures were developed, in which the relevant information were presented with geographical location (in some cases) and relevant statistical information reported where possible. The search yielded 1,223 articles, out of which a total of 167 publications were relevant to this review based on the inclusion criteria described in section Inclusions and Exclusion Criteria. This number comprises 156 articles, 2 book chapters, 5 relevant guidelines and four documents. Out of these publications, 50 were focused on human health risks from partially treated and untreated wastewater reuse, 19 linked reuse to specific infection and approximately 100 publications indirectly assessed the health risks from wastewater reuse. With wastewater reuse as written in the rest of the paper we include partially treated as well as untreated wastewater, if anything else is not stated. In the relevant sections the type of wastewater is stated to give clarity and aid in comparison.

## Results

### Occurrence of Pathogens in Wastewater Used for Irrigation

**Figure 1:**
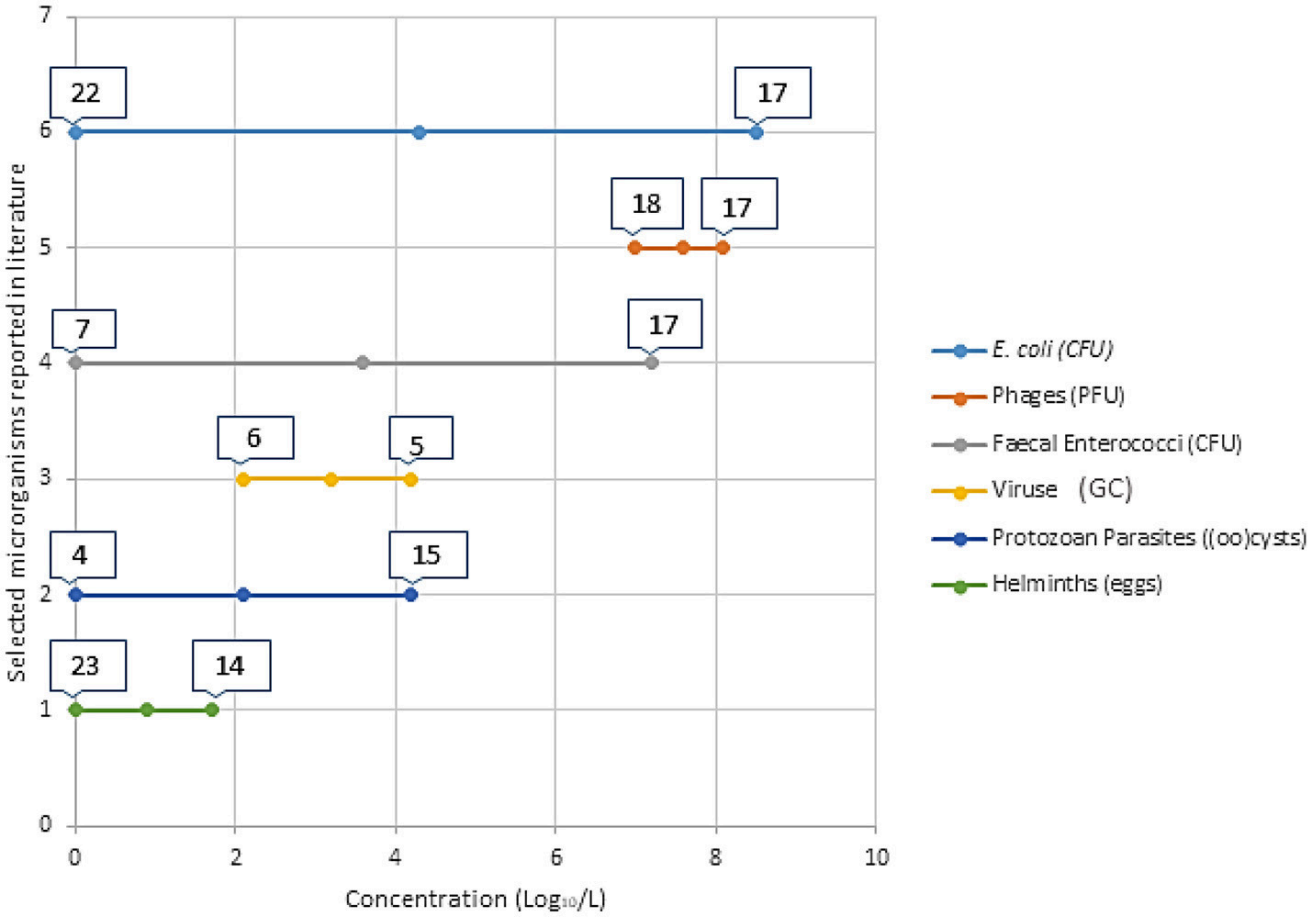
Visual representation of minimum and maximum concentration (Log_10/_L) of selected microorganisms in partially treated and untreated wastewater intended for reuse. The numbers on the bars represents the individual references with these reported concentrations. These articles are referenced in Table 1, where further information is provided. This is a brief introduction of the different concentrations reported in literature for the different categories of microorganisms. Table 1 gives detailed information on the literature added in this figure.

Health risks associated with partially treated and untreated wastewater reuse are dependent of exposure combined with the presence and concentrations of hazards (e.g., pathogens). The excreted concentrations of pathogens (i.e., the hazards) vary based on the pathogen type and strain, the affected individual, and the phase of the infection cycle, and are thus dependent on the health of the population and the resilience of the pathogens to environmental stressors (35, 36). Figure 1 gives a general background graphical representation of the concentration of selected microbial indicators and pathogens reported in the literature. Detailed data from publications is presented in Table 1.

**Table 1:** Concentration of fecal indicator organisms and pathogens reported in selected literature from different types of wastewater used for irrigation.

References^*^	**Pathogen or fecal indicator**	**Concentration (original values transformed to Log_10_/L)**	**Type of Wastewater**	**Country**
(37)	*E. coli*	1.8–2.8 (CFU)	Secondary and tertiary treated wastewater	Italy
	Fecal coliforms	2.5–3.0 (CFU)	
	Fecal enterococci	2.1–2.2 (CFU)	
(4)	Fecal coliforms	7.89–8.15 (CFU)	Untreated wastewater	Mexico
	Fecal enterococci	6.71–7.18 (CFU)	
	*Cryptosporidium parvum*	2.17–2.48 (Oocyst)	
	*Giardia lamblia*	2.43–2.69 (cyst)	
	*E. coli*	7.0–7.49 (CFU)	
	*Klebsiella pneumoniae* (Carbapenemase resistant)	5.28–5.98 (CFU)	
	Vancomycin resistant enterococci	4.0–5.77 (CFU)	
(38)	*E. coli*	2.1–5.0 (CFU)	Wastewater contaminated surface water	Spain
(39)	*E. coli*	3.95–4.32 (MPN)	Treated wastewater	Italy
	*C. perfringens*	5.69–5.81 (CFU)	
	Somatic coliphages	5.18–5.23 (PFU)	
	*Giardia lamblia*	1.46–1.56 (Cysts)	
	*Cryptosporidium parvum*	0–1.34 (Oocysts)	
(40)	Norovirus GII	3.5–4.0 (GC)	Treated wastewater	France
	Norovirus GI	3.5–4.2 (GC)	
	Rotavirus	4.0 (GC)	
(41)	human adenovirus (HAdV)	2.94–3.26 (GC)	Treated wastewater	Australia
	human polyomavirus (HPyV)	2.11–2.65 (GC)	
	human torque teno virus (HTtV)	2.85–3.38 (GC)	
	Microviridae	2.36–3.30 (GC)	
(42)	*E. coli*	0–4.0 (CFU)	Treated wastewater	China
(43)	*E. coli*	0–5.89 (CFU)	Treated wastewater	Italy
	Enterococci	0–5.44 (CFU)	
	Sulphite-reducing *Clostridium* spores	1–5.49 (CFU)	
(44)	Fecal coliforms	3.96 (CFU)	Treated wastewater	Israel
	*E. coli*	3.29 (CFU)	
	Enterococcus	3.51 (CFU)	
(45)	*Cryptosporidiumparvum*	0–1.09 (Oocysts)	Treated wastewater	China
	*Giardia lamblia*	0–2.02 (Cysts)	
(46)	Fecal coliforms	< 1.30 (MPN)	Treated wastewater	Jordan
(47)	*E. coli*	3.81–4.47 (CFU)	Treated wastewater	Italy
(48)	*E. coli*	2.9–6.7 (CFU)	Wastewater contaminated surface water	Brazil
(49)	*Ascaris lumbricoides*	1-1.78 (Eggs)	Treated and untreated wastewater	India
	*T. trichiura*	0–0.30 (Eggs)	
	Hookworm	0–0.95 (Eggs)	
(50)	*Cryptosporidium parvum*	0–1.04 (Oocysts)	Treated wastewater	Spain
	*Giardia lamblia*	0–0.78 (Cysts)	
(51)	*Giardia lamblia*	< 1.72–4.24 (Cysts)	Treated wastewater	Costa Rica
		< 0.76–3.95 (Cysts)		Mexico
		< 1.26–3.26 (Cysts)		Panama
		< 0.89–1.60 (Cysts)		USA
	*Cryptosporidium parvum*	< 1.72–2.52 (Oocysts)		Costa Rica
		< 0.76–3.19 (Oocysts)		Mexico
		< 1.26–2.39 (Oocysts)		Panama
		< 0.89–1.63 (Oocysts)		USA
(52)	Thermotolerant coliform	7.0–10.0 (MPN)	Wastewater contaminated surface water	Ghana
	*E. coli*	5.2–8.5 (CFU)	
	*Enterococci*	4.3–8.1 (CFU)	
	Somatic coliphages	3.6–6.9 (PFU)	
	F+ coliphage	3.3–5.7 (PFU)	
(53)	*Cryptosporidium parvum*	< 1.23–2.30 (Oocysts)	Surface water/wastewater	Mexico
	*Giardia lamblia*	< 1.23–3.21 (Cysts)	
(54)	*E. coli*	0.5 (MPN)	Type 2 reclaimed water	USA
	Total coliphage	2.1(PFU)	
	*C. perfringens* (total)	0.60(CFU)	
	Norovirus GII	2.4(GC)	
	Adenovirus A-F	2.7(GC)	
(55)	Fecal coliforms (100 ml)	6.7 CFU	Treated wastewater (conventional activated sludge)	Iran
(56)	*E. coli*	6.2 CFU	Treated wastewater	South Africa
			
(57)	*E. coli*	0 CFU	Treated wastewater	Spain
(58)	Fecal Streptococci	1.7–2.6 CFU	Treated wastewater	Morocco
	Helminths	0 Eggs/L	

* *Numbers before the reference refer to indication in Figure 1*.

*GC, Gene copies; MPN, Most Probable Number; CFU, Colony Forming Units; PFU, Plaque Forming Units*.

### Exposure Pathways

Farmers and farm workers may be directly exposed to pathogens, and consumers of the farm produce will be indirectly affected. Additionally, the surrounding community can be further affected through the contamination of groundwater and run-off to surface water (59). Aerosols may also be formed in farms were sprinkler irrigation is practiced, affecting nearby communities as well (60, 61).

Different exposure pathways have been implicated in the spread of diseases associated with reuse of wastewater, especially partially treated and untreated, with examples shown in Table 2. The main route through direct contact could be termed as an occupational exposure pathway. This has been implicated in the spread of diarrhea (2, 4, 62, 64), parasitic infections (63, 67, 68) and skin infections (5, 6, 22)]. The amount of direct contact with wastewater during its application is largely dependent on the type of irrigation practiced and individual behavior. For example, the use of watering cans for the collection of irrigation water from ponds, as is the case in many developing countries where the practice of wastewater irrigation is common, results in the greatest exposure (76). This exposure leads to greater risk due to the non-use of personal protective equipment like boots and nose masks. Furrow or flood irrigation also increases the possibility of direct contact with the wastewater, increasing risks of infections (18, 77).

**Table 2:** Exposure used in the determination of diseases associated with wastewater irrigation from selected literature.

**Location**	**Health risks**	**Route of exposure**	**Type of wastewater**	**Authors**
Mezquital Valley, Mexico	Diarrhea	Occupational exposure, aerosols exposure to resident, underground water contamination	Untreated wastewater	(4)
Uppsala, Sweden	Gastroenteritis (rotavirus-based)	Direct ingestion of greywater during maintenance	Treated greywater	(62)
Vietnam	Parasitic infection (*Ascaris lumbricoides* and *Trichuris trichiura*	Occupational exposure and consumption of vegetable	Partially treated and untreated wastewater	(63)
Brazil	Gastrointestinal infection (*E. coli* and rotavirus)	Consumption of salad crops	Partially treated wastewater	(64)
Bangkok, Thailand	Diarrhea (*Giardia lamblia* and *Entamoeba histolytica*)	Direct exposure	Untreated wastewater	(2)
Thailand and Canada	Gastroenteritis	Swimming, fishing, consuming canal water-irrigated vegetables, and ingesting/inhaling water or aerosols while working in canal water-irrigated fields	Wastewater contaminated Surface water	(65, 66)
Malamulele, South Africa	Parasitic infections (hookworm and *G. lamblia*)	Exposure via occupational consumption	Partially treated wastewater	(67)
Phnom Penh, Cambodia	Skin infection	Occupational exposure	Partially treated wastewater	(7)
Musi River, India	Skin infection/irritation	Exposure to infected source	Partially treated wastewater	(20)
Hyderabad, India	Intestinal parasitic infection	Occupational exposure	Partially treated and untreated wastewater	(68)
Vietnam	*Escherichia coli* infection (risk)	Occupational exposure	Untreated wastewater	(69)
Hanoi, Vietnam	Skin infection	Occupational exposure	Partially treated wastewater	(22)
Hanoi, Vietnam	Diarrhea	Children of occupationally exposed farmers	Partially treated wastewater	(70)
Faisalabad, Pakistan	Giardiasis	Occupational exposure	Untreated wastewater	(71)
Vietnam	Helminthic infection	Occupational exposure	Untreated wastewater	(72)
Nghe An Province, Vietnam	Helminthic infection	Occupational exposure	Partially treated wastewater	(73)
Marrakech, Morocco	Infection of *Ascaris, Trichuris*	Children resident in wastewater irrigated farmhouse	Untreated wastewater	(74)
Vietnam	Intestinal parasitic infection	Occupational exposure	Unknown	(75)

*Occupational exposure refers essentially to farmers*.

The use of sprinkler irrigation may also lead to greater exposure not only for farmers and other farm workers but for the general community because of exposure to aerosols (78–80). Communities living close to wastewater irrigation sites in Israel where sprinkler irrigation was practiced had an IRR of 1.08 for diarrhea, although this risk of infection was lower than the risk from direct exposure to partially treated and untreated wastewater (IRR = 1.23). That study particularly showed the impact of sprinkler irrigation on the health of a larger population (60). Aerosols may be inhaled or may settle on food (81). Aerosols from wastewater are also reported to contain several different types of pathogens. Li et al. (82) showed that the suspension of *Actinomycetes* (aetiological agent for actinomycosis) and other pathogenic microbes in aerosols from partially treated wastewater constitute health hazard in Xi'an, China.

Consumption of produce, especially vegetables, from wastewater irrigated farms poses an additional pathway for transmission of pathogens from wastewater to the general public. Agunwamba (83) demonstrated that consumers of vegetables from wastewater irrigated fields leads to an IRR of 1.75 for diarrhea. Section Food-Borne Outbreaks (Human) Associated With the Consumption of Fresh Produce Irrigated With Wastewater discusses this exposure pathway in detail.

#### Food-Borne Outbreaks (Human) Associated with the Consumption of Fresh Produce Irrigated with Wastewater

Bacterial concentrations in wastewater and air quality around the farmyard have been shown to closely relate. A study by Teltsch and Katzenelson (84) detected aerosolized coliforms with concentration ≥10^3^ CFU/mL. The pathogens carried may depend on their size and the force of the spray. Small sized pathogens are more commonly transported during irrigation (85, 86). Enteric viruses may occur in significant concentration in aerosolized form, when compared with the varieties of microorganisms found in wastewater (85). Their retention in wastewater for irrigation is usually due to the inability of some WWTPs to effectively achieve virus removal. It also depends on high resistance to environmental stress by some of these viruses. For aerosolized bacteria, relative humidity and solar irradiation may affect their viability and subsequently their concentration in the air. The aerosolized bacterial pathogens may also be affected by UV, as there was reports of 10 times more aerosolized bacteria measured during night irrigation than with day irrigation (84).

The concentrations of pathogens that reach farmyard houses and lead to exposure in consuming food and inhalation may depend on the wind velocity (87). The probability of initiating infection also depends on the infective dosage. For example, low quantities of viruses (10^2^ pfu), such as for noroviruses, are enough to initiate infection through the respiratory pathway (85). Depending on the irrigation methods (i.e., sprays or sprinklers), farm workers and community members in the surrounding area may be exposed to the aerosolized pathogens from the wastewater (88, 89).

Aerosol spray may also facilitate deposition of contaminated wastewater on the edible parts of the crop. Surface contamination of these has been reported as a major means of transmission of diseases through consumption (90–93). Additionally, internalization of pathogens in different vegetables has been documented (94–98), which could be another route of transmission of these pathogens to the consumers. The re-growth of these pathogens on vegetables has also been reported (99) and is partly influenced by factors such as, their adhesion ability to the plant surfaces (100, 101) and the persistence of the particular pathogen (102, 103).

Globally there have been different reported outbreaks, for example 11 lettuce-related outbreaks of gastroenteritis with a total of 260 reported cases were reported in Denmark (104). Researchers emphasize that adhesion of pathogens to surfaces and internalization of pathogens placed high demand of responsibilities on consumers to avoid gastroenteritis (105). Most outbreaks have been attributed to infection with different strains of *Salmonella* (106–109), Enterohemorrhagic *E. coli* (110–113), and viruses such as norovirus (114). Table 3 presents examples of food-borne human outbreaks (microbial) associated with the consumption of fresh produce irrigated with wastewater. The type of irrigation practice plays a critical role in the contamination of farm produce. A study by Makkaew et al. (121) assessed the contamination of *E. coli* in lettuce grown under four different methods of wastewater irrigation; open spray, spray under plastic sheet cover, open drip, and drip under plastic sheet cover. *E. coli* contaminations were detected in all the lettuce samples with all types of spray beds. Submersed lettuce irrigated with wastewater contained 1,300 *E. coli* MPN/100 mL and had equal levels of contamination as with the spray irrigation. The scenario was similar in both laboratory and experimental investigations (121). The study also showed that crops are seriously imparted by wastewater irrigation, independent of the irrigation schemes used (121). In a study on the use of wastewater in agriculture, Antwi-Agyei (122) identified irrigation with partially treated and untreated wastewater as a key risk factor for the observed contamination of 80% of produce samples. The study reported the median concentration ranging from 0.64 to 3.84 log_10_ *E. coli*/g produce, and fresh salad having as high as 4.23 log_10_ *E. coli*/g. In a similar study, Bouwknegt et al. (123) estimated the following risks per serving of lettuce based on the QMRA models: 3 × 10^−4^ (6 × 10^−6^ −5 × 10^−3^) for NoV infection and 3 × 10^−8^ (7 × 10^−10^ −3 × 10^−6^) for hepatitis A jaundice. In that study however, the risk of wastewater irrigation was less than the effect of hand's contact (123).

**Table 3:** Examples of food-borne outbreaks (human) associated with the consumption of fresh produce irrigated with wastewater from selected literature (Only the reports with year of outbreak, food implicated and number of cases were considered).

**References**	**Country**	**Pathogens**	**Outbreak year**	Food implicated^*^	**No of cases**
(115)	China	*Salmonella paratyphi* A	2010–2011	Consumption of raw vegetables	600
(106)	Unspecified	*Salmonella saintpaul*	2008	Jalapeño peppers, serrano peppers, tomatoes	1,442
(116)	Sweden	Enterohemorrhagic *E. coli*	2013	Fresh salad	19
(104)	Denmark	Noroviruses and enterotoxigenic *Escherichia coli*	2010	Lettuce of the lollo bionda type	260
(117)	Norway	*Salmonella* species	2004; 2006; 2007	Rucola Lettuce	21
(112)	Sweden	*E. coli* 0157	2005	Iceberg lettuce	135
(114)	Denmark	Norovirus	2005	Raspberries	>1,000
(118)	Canada	*Cyclospora cayetanensis*	2011	Basil	17
(119)	Germany	*Cyclospora cayetanensis*	2000	Green vegetables	34
(120)	Saudi Arabia	Hepatitis A virus	1996	Food (unspecified); specified not linked	94

* *Grown with contaminated (fecal) water*.

#### Impact of Risk Awareness on Exposure

Risk awareness might also be a factor that includes handling of the crops and vegetables irrigated with wastewater. Antwi-Agyei et al. (105) reported that knowledge of the irrigation water source and associated quality were associated with higher awareness of health risks (OR = 4.6, *p* = 0.06). However, the outcome was not impacted by demographic factors like age, education, or gender (OR = 4.7, *p* = 0.12). A higher awareness of health risks may change on-farm practices and also reduce the exposure of farmers to the pathogens in the wastewater, as well as, reduce the concentration of pathogens on the farm produce leading to lesser adverse health impact on the both farmers and consumers. Reuse of wastewater in aquaculture has also been linked with adverse health effects (124). For instance, fish farmers without understanding of health risk and protective clothing were found to have a high exposure to the pathogens in the wastewater, leading to skin infections (21).

### Epidemiological Evidence of Human Health Risks

The different types of infections reported for people exposed to partially treated and untreated wastewater used in agriculture are discussed in the following sections. Section Wastewater Reuse, Diarrhea and intestinal infection summarizes the epidemiological evidence of diarrheal and other intestinal infections. Section Wastewater Reuse and Intestinal Parasitic Infections summarizes the evidence of intestinal parasitic infections and section Skin Infections the evidence of skin infections.

#### Wastewater Reuse, Diarrhea, and Intestinal Infection

**Figure 2:**
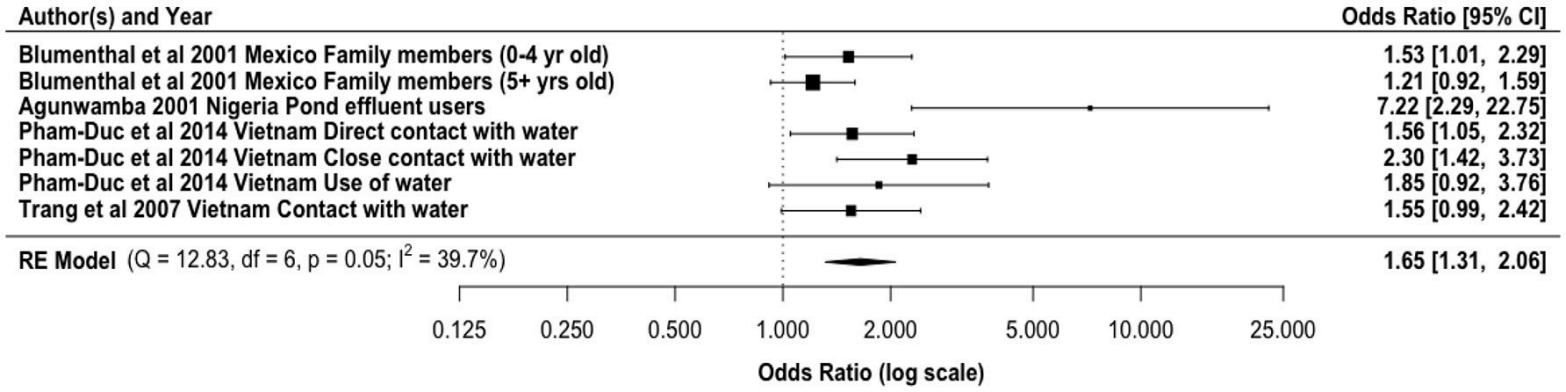
Odds ratio for exposure to partially treated and untreated wastewater and diarrheal disease incidence from selected literature.

Partially treated and untreated wastewater reuse has been directly linked with diarrheal diseases (Figure 2). Our meta-analysis gave an overall odds ratio of 1.65 (95% CI: 1.31, 2.06) for diarrheal diseases among agricultural workers and family members exposed to wastewater for irrigation. Direct exposure by farm workers to wastewater has been shown as the main transmission route. However, farm workers may also be exposed to pathogens based on their contact with soil that has been contaminated by wastewater, especially partially treated and untreated. In addition to the normal microflora, wastewater irrigated soils have elevated pathogen concentrations. For example, Klutse (125) reported higher concentrations of *Enterobacteriaceae, Bacillus* spp., *Staphylococcus* spp., *Pseudomonas* spp., and *Clostridium* spp. in irrigated soils. Antwi-Adyei et al. (126) identified soil as the main risk pathway with factors such as working barefooted (93% of farmers), hand contact with contaminated soil (86% of the farmers), and contaminated hands to mouth (53% of the farmers) as the main contributors. Additionally, the regrowth of bacteria in the environment may contribute to the increased incidence of diarrhea, as the high nutrient content of wastewater can enhance bacterial regrowth (127, 128). Modeled risks based only on pathogens contained in the liquid effluent from wastewater treatment plants, without accounting for regrowth, may lead to the underestimation of risks.

The majority of the epidemiological studies on reuse and diarrheal diseases have focused on the direct exposure to the wastewater especially by the farmers and farm workers. However, Shuval et al. (60) demonstrated that aerosols could lead to a diarrheal incidence rate ratio of 1.08 for people living close to irrigation fields in Israel. Living in a household with someone engaged in untreated wastewater reuse could also result in higher risk of diarrhea (OR = 2.69) (21). This relate to the possibility of additional human to human transmission within the home. This is important because domestic hygiene has been implicated in the increase in diarrheal diseases within the home setting for children (129, 130). Within the domestic domain several other sources of contamination, like from food, contaminated drinking water in the home (131) or direct exposure to feces from humans and animals will superimpose on the effects that is due to the irrigation with wastewater with linked exposure.

Children and immunocompromised individuals have been shown to have higher risks of diarrheal infections than immunocompetent adults (132–135). Blumenthal et al. (77) reported an increase in the risk of diarrhea (OR = 1.75) among children aged <5 years. This diarrhea may be from the ingestion of pathogenic microorganisms, such as *Salmonella* species and Diarrheagenic *Escherichia coli* (2, 70, 77, 136) in either the soil or wastewater.

#### Wastewater Reuse and Intestinal Parasitic Infections

**Figure 3:**
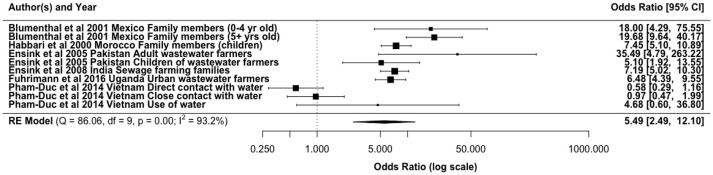
Odds ratio for exposure to partially treated/untreated wastewater and helminth infections from selected literature.

Intestinal parasitic infections, especially soil-transmitted helminths, are reported as the major health concern associated with wastewater reuse (34). This is partly due to their high persistence in the environment. In line with this concern, epidemiological studies have shown an increase in parasitic infections from the use of wastewater (Figure 3). The main exposure route accounted for in these studies has been direct contact for farmers and other farm workers, where increased risks with ORs 0.58–3 have been reported (3, 19, 137, 138). In a study conducted in urban and peri-urban transition zones in Hanoi, Vietnam, Fuhrimann et al. (139) reported that peri-urban farmers had the highest adjusted odds of acquiring intestinal parasitic infection among various groups considered (OR 5.3, 95% CI: 2.1–13.7). However, Trang et al. (72) established that wastewater reuse did not pose significant risks for helminth infections among rice farmers using wastewater in Vietnam, although untreated wastewater is used. Our meta-analysis gave an overall odds ratio of 5.49 (95% CI: 2.49, 12.10) for helminth infections in agricultural workers and family members exposed to wastewater for irrigation (Figure 3).

The infection risks may differ between the types of parasitic pathogens found in wastewater due to variations in concentration and the mode of exposure to the irrigation water. There is however similarities in the consistency of found values. For instance, in Ghana risks of infection with *Ascaris* and hookworm was found among the farmers and family member exposed to irrigation water (surface water contaminated with untreated wastewater) to be similar with ORs of 3.9 (95% CI, 1.15–13.86) and 3.07 (95% CI, 0.87–10.82), respectively (3). Pham-Duc (19) reported *Ascaris* infection OR of 2.1 (95% CI 1.4–3.2) vs. an OR of 1.5 (95% CI 1.0–2.3) for *Trichuris trichiura*. A cross-sectional survey of children exposed to wastewater irrigation in Morocco showed a 20.5% higher prevalence of *Ascaris* infection compared to 3.8% for unexposed children (18). From the same study in Morocco the difference in *Trichuris* infection among the exposed (0.4%) and unexposed (0.3%) children was reported to be non-significant. Furthermore, approximately 45% of farmers using wastewater effluents for irrigation in Asmara villages, Eritrea were infected with giardiasis, with consumers of their vegetables 7% more likely to be infected with giardiasis than a control group (140).

These differences and similarities in infection prevalence from the exposure could be attributed to (1) the difference in the concentration of the infectious eggs in the wastewater, which is dependent on the type of wastewater treatment, (2) differences in their persistence in soil or on crops, or (3) the efficiency with which they are transferred from irrigation water to soil/crops. For example, Verbyla et al. (10, 141) reported higher ratios of helminth egg and parasitic (oo)cyst (*Giardia* and *Cryptosporidium*) concentrations on irrigated soil and crops relative to their concentrations in wastewater used for irrigation, with lower corresponding ratios for coliphages and for the viral fecal indicator pepper mild mottle virus. The parasite eggs and (oo)cysts may be more efficiently transferred from irrigation water to crops/soil than some viruses, and/or it supports previous findings that the parasites persist longer on crops or in soil than viruses (142). Additionally, pathogen concentrations in wastewater are also a reflection of the infection levels in the populations. Hookworms in particular remain a major cause of morbidity in developing countries, which are accompanied by gastrointestinal symptoms and chronic anemia (143). This epidemiological evidence therefore shows a linkage or interconnectedness of infection and pathogen concentration in wastewater.

The risk of parasitic infections from either direct or indirect exposure to wastewater also will vary depending on factors, such as age, gender, frequency of exposure etc. For instance, Blumenthal et al. (77) reported a close association between direct exposure to untreated wastewater and an excess risk of *A. lumbricoides* infection in children aged < 5 years (OR = 18.0). Also, a cross-sectional survey of people aged ≥ 18 years at risk to wastewater exposure showed a 30% point-prevalence of intestinal parasitic infections (139).

#### Skin Infections

Exposure to wastewater could result in different types of skin infections, such as, dermatitis, urticarial, fungal infections of toe- or fingernails (23). Trang et al. (5, 6) suggested that the skin diseases may be deep systemic dermatitis and other fungal infections. Studies conducted in Musi River, India; Phnom Penh, Cambodia, and Hanoi, Vietnam showed skin infection and irritation as the major effect of the exposure (7, 22). However, only a few studies have focused on an epidemiological link. For example, a cross-sectional survey in Vietnam involving over 235 farmers using wastewater for irrigation or aquaculture reported an OR for dermatitis of 3.0 (95% CI: 1.1–7.7) (22). A similar study from the same country reported a lower OR of 2.7 (95% CI: 1.3–5.8) for direct contact with wastewater (6). In addition to direct contact, living in a household with someone involved in wastewater reuse increased the risks of skin infections (5). Frequency of exposure, immunological status, occupation and the lack of protective coverings were identified as the determining factor predisposing individuals to this effect of wastewater (5, 144).

### Estimation of Health Risks Associated with Wastewater Reuse Using Quantitative Microbial Risk Assessment (QMRA)

Quantitative Microbial Risk Assessment (QMRA) is a modeling process that estimates the potential human health risks from exposure to different pathogens (e.g., human pathogenic viruses, protozoa, and bacteria) (145) especially through food and/or water (146). This is a structured approach that integrates information and data with mathematical models to examine exposure and spread of microbial pathogens and in the process characterize the human health risks (147, 148). QMRA has been used extensively in the risk estimation of infections for different exposed groups related to wastewater reuse (25, 29, 31, 149, 150). The risk estimates from these studies vary, mainly due to difference in the concentration, for example by viable STH eggs ingested by exposed populations. QMRA has been used by the WHO (34, 151) and the Australian government (152, 153) to determine pathogen reductions needed to achieve a health target of 10^−6^ DALYs per person per year (ppy) (154).

The use of QMRA allows for the quantification of infection risks for different populations that has ingested or been exposed to different concentrations of pathogen. The exposure could be either from intentional or accidental ingestion of wastewater, consumption of farm produce or ingestion of groundwater from irrigation fields etc. Assuming ingestion of wastewater and soil, Seidu et al. (31) showed that two farmers out a hundred using wastewater for irrigation were likely to be infected with *Ascaris* due to accidental ingestion of the irrigation water. However, accidental ingestion of the farm soil may result in infection of all exposed farmers, possibly due to accumulation of the parasitic eggs in the soil. Considering risks from inhalation of aerosols, Courault et al. (40) estimated norovirus infection risks of >10^−4^. Carlander et al. (88) reported high risks of rotavirus infections in Culmore, Northern Ireland [P(inf) 8 × 10(−1)] and Kvidinge, Sweden [P(inf) 7 × 10(−1)] when wastewater is used for irrigation. Estimation of infection risks from aerosols using QMRA, generally takes into account the wind speed and distance of the exposed population.

QMRA estimation of infection risks could also account for different crops. This is important especially in relation to the amount of irrigation water that could possibly remain on the crops by the time of harvest and subsequent consumption. While considering different wastewater-irrigated vegetables, Mok et al. (155) observed that consumption of lettuce posed the greatest health risks as compared to cucumber and broccoli. The researchers reported that the median annual norovirus disease burden across the different vegetables ranged from 7.95 × 10^−5^ to 2.34 × 10^−3^ DALY/person/year (155).

Risk estimates produced by QMRA are dependent on the input dose or concentration of pathogen used among other factors such the dose response model used and frequency of exposure. The concentration of the pathogen could be either determined in the laboratory or assumed. Some researchers use fecal indicator microorganisms to estimate pathogen concentrations in wastewater and thereafter determine the infection risks. This is mainly due to the ease of detection of these microorganisms, however the survival and even removal/inactivation of these in the environment differs greatly from many pathogenic microorganisms. It was observed by Owusu-Ansah et al. (156) that the use of fecal indicator conversion ratio model to estimate health risks results in the underestimation of the risks involved. Using improved methods of detection, Cutolo et al. (25) reported average densities of 1 cysts L^−1^ and 6 eggs L^−1^ for *Entamoeba coli* and *Ascaris* spp., respectively. The reported annual risks of *Ascaris* infection resulting from accidental ingestion of wastewater irrigation were therefore reported to be 7.5 × 10^−2^ in 208 days and 8.7 × 10^−2^ in 240 days (25).

### Emerging Contaminants

#### Antibiotics and Antibiotic Resistance Genes (ARGs) in Wastewater for Reuse

Antibiotic residues and antibiotic resistant genes ARGs have been reported in wastewater released from wastewater treatment plants (157–162). The presence of these in wastewater has been identified as a possible source of antibiotic resistance microorganisms (13, 163). This resistance could be developed through induction, selection or horizontal gene transfer of ARGs (162, 164). Varying antibiotic removal efficiencies from wastewater for reuse has been reported (165–167).

These studies show that most treatment plants have antibiotics in their effluents which when reused may impact and accumulate in soil and be taken up by the crops. Reported concentration of antibiotics and antibiotic resistance genes identified in treated wastewater are exemplified in Table 4. Negreanu (185) assessed the presence of antibiotic-resistant bacteria and antibiotic resistant genes in agricultural soils irrigated with wastewater and Broszat et al. (186) concluded that wastewater irrigation increases abundance of potentially harmful Gamma-proteobacteria with antibiotic resistance genes in soils from Mezquital Valley, Mexico. These studies suggest the emergence of antibiotic resistance as future potential concern for reuse of wastewater.

**Table 4:** Reported concentration (from selected literature) of antibiotic residues and antibiotic resistance genes in partially treated and untreated wastewater for reuse and the irrigated soil.

**References**	**Country**	**Antibiotics/ARGs**	**Excretion rate (% of the administered dose excreted)**	**Concentration (ng/L) of residual antibiotics**
(168)	China	Clarithromycin	30.0–40.0 (25.0^*^)	0.3–115.0
		Azithromycin	6.0–12.0	1.9–287.5
		Erythromycin-H_2_O	2.0–15.0	13.2–338.7
(169)	Poland	Sulfamethoxazole	>50.0 (30.0^*^)	857–1,149
				1,021–1,431
		Trimethoprim	>60.0 (80.0^*^)	340–398
				234–332
		Erythromycin	2.0–15.0	14–18
				15–19
(170)	Portugal	Sulfonamides	>50.0	400–800
				2,600–2,800
(171)	China	Cefalexin	?80.0	170–5,070
		Amoxicillin	60.0	64–1,670
(172)	China	Cefalexin	80.0	240–1,800
		Erythromycin	2.0–15.0	470–810
		Tetracycline	>60.0	96–1,300
(173)	Australia	Amoxicillin	60.0	6,940
(174)	Hong Kong	Ciprofloxacin	50.0–70.0 (20.0^*^)	720
(175)	Italy	Ofloxacin	65.0–80.0	600
(176)	Germany	Erythromycin	2.0–15.0	2,500–6,000
(177)	Brazil	Penicillin G	60.0–90.0 (parenteral)	434,460
			UA (Enteral)
**SOIL (S)/GROUNDWATER (GW) IMPARTED BY WASTEWATER IRRIGATION**					**S or GW**	**(NG/KG FOR SOIL)**
(178)	Cyprus	Sulfamethoxazole	S	980
		Trimethoprim		620
(179)	China	Tetracycline	S	69.3–234.0
		Sulfamethoxazole		4.0–58.2
(180)	USA	Trimethoprim	GW	1,000
(181)	USA	Ofloxacin	GW	19.3–604.9
		Nalidixic acid, Erythromycin, Clarithromycin and Azithromycin		26.9–453.2
**ANTIBIOTIC RESISTANCE GENES (ARGs) GENE COPIES/ML**				
(157)	Romania	*sulI*		5.33 × 10^2^-1.94 × 10^1^
		*qacEΔ1*		1.94 × 10^2^-4.89 × 10^2^
		*blaSHV*		1.69 × 10^3^-4.39 × 10^3^
		*mefA*		1.47 × 10^3^-1.67 × 10^3^
		*catA1*		6.83 × 10^5^-2.63 × 10^3^
^#^(182)	India	*blaTEM*		1.3 × 10–1.02 × 10^4^
(158)	China	*tetG*		2.30 × 10^7^
		*floR*		4.37 × 10^7^
		*sul2*		1.60 × 10^8^
(183)	Saudi Arabia	*tetO*		2.5 × 10^2^
		*tetQ*		1.6 × 10^2^
		*tetW*		4.4 × 10^2^
		*tetH*		1.6 × 10^1^
		*tetZ*		5.5 × 10^3^

*sulI gene codes for resistance against sulphonamides; qacEΔ1 gene codes for resistance against quaternary ammonium compounds; mefA gene codes for resistance against Macrolide-lincosamide-streptogramin B antibiotics; catA1 gene confers resistance to chloramphenicol. UA = unavailable*;

* *Verlicchi et al. 184*;

# *Measurement was in gene copies per 100 mL*.

The concentration of antibiotics will be reduced during treatment and the efficiency of removal is dependent on the treatment technology (187, 188) and the physical state of the antibiotic compound (189). Varying removal efficiencies of different antibiotics have been reported in the USA and China (165, 172, 190–192). All studies reported that residual antibiotics persist in treated wastewater and sludge in most cases and can be conveyed along the reuse pathways.

#### Fate of Antibiotics and Antibiotic Resistance Genes (ARGs) in Wastewater

Some antibiotics remain intact in wastewater after excretion, while others may form complexes or change the parental configuration. This may however be reconverted into their parent compounds by deconjugation during biological treatment (193). Due to their hydrophobicity some of the antibiotics may also be sorbed to particles. Based on the removal of particles during wastewater treatment, the aqueous concentration of antibiotics will change but they will be transferred to sludge. Some may also be desorbed with intact configuration and appear in the final effluents together with hydrophilic ones (194). Norfloxacin and tetracycline exemplifies sorption to particulate matters, which can be highly variable.

When the wastewater is used for irrigation on farmland the residual antibiotics and ARGs may impact the normal microflora of the soil (157–162, 164). Their persistence in soil depends on factors that includes sorption to soil particles that also influences leaching potential to groundwater and their biotic and abiotic degradation. The most essential abiotic reactions are photochemical and chemical mediated transformations, which may include hydrolysis, oxidation and reduction, which would occur in upper soil layers (195). One research report by Pan and Chu (196) postulate that tetracycline possesses a higher sorption affinity in agricultural soil when compared with quinolones, macrolides, chloramphenicol, and sulphonamides and sulphonamides were reported to have high leaching potential to the groundwater (197).

#### Uptake and Internalization of Antibiotics and ARGs by Edible Crops

The antibiotics in wastewater for irrigation will impact the soil directly and may result in eco-toxicological effects (198). The effects of wastewater irrigation on the occurrence of ARGs were reported by Jechalke et al. (199) over a period of 100 years of irrigation. Because the nutrients in the wastewater support regrowth of some bacterial pathogens the presence of sub-inhibitory concentration of the antibiotics can bring about resistance by induction and/or expression of ARGs taken up.

Christou et al. (178) reported an uptake of sulfamethoxazole and trimethoprim by tomato crop plants with fruit bio-accumulating 5.26 μg kg^−1^ of sulfamethoxazole and 3.40 μg kg^−1^ of trimethoprim. The antibiotics, ARGs and the antibiotic resistant pathogens (ARP) may be taken up (like nutrients). The ARP may pass through the natural root apertures into the xylem or phloem of the plants, be translocated and internalized in edible part of the crop (200–203). By this they may impact the consumers (204) especially when the crops involved are to be eaten raw (200, 204). For these categories of uncooked crops, certain bacterial species like *Stenotrophomonas maltophilia* and *Acinetobacter* species usually found in wastewater and root rhizosphere can be a threat if allowed to contaminate the crop (14, 205). This is because they are also known for being reservoirs of wide range of ARGs, extreme drug resistance and as etiological agent of life threatening infections (14, 206, 207).

These ARP might be difficult to treat due to their antibiotic resistance status. Carey et al. (208) detected total enterococci and vancomycin resistant Enterococci in 71% (34/48) and 4% (2/48) of reclaimed water samples, respectively with *Enterococcus faecalis* as the most prominent species. These Enterococci in the reclaimed water exhibited high antibiotic resistance at the point of water use as measured using their minimum inhibitory concentrations (MICs). Contaminated water used for irrigation may initiate common source epidemics as in the outbreak of food borne pathogen in some countries in Europe as reviewed by Okoh et al. (209).

Apart from ARP, antibiotics in the wastewater cause phytotoxicity. A screening for the level of phytotoxicity by antibiotics internalized into seedlings and plants was done using seed germination experiment by Hillis et al. (210) and Pan and Chu (196) as well as plant growth tests (211). The results showed varying toxic effects of antibiotics on the cultivars. Pan and Chu (196) showed that the antibiotics were taken up and it inhibited root elongation significantly (*p* < 0.05). Tetracycline was noted to have the highest level of toxicity among the antibiotics while lettuce crop was found to be the most affected by larger number of the veterinary antibiotics (212).

#### Risk Assessment of Antibiotics in Reused Wastewater

Residual antibiotics and ARGs remain two major concerns in integrated human health risk assessment associated with the use of wastewater (213–215). The risk associated with reuse of wastewater containing antibiotics or any pharmaceutical is generally expressed as risk quotient (RQ) or hazard quotient (HQ). The risk quotient (RQ) for antibiotic in reused wastewater reflects the extent of associated ecological risks (216). RQ, which is a calculated index, constitutes a vital analytical tool for assessing ecotoxicological risks (217, 218). It varies proportionally with the detected concentration of the antibiotics in the wastewater. It is the ratio of measured concentration (MEC) in wastewater to the predicted no effect concentration where no effect concentration (PNEC) as follows:

(1) $$\begin{array}{r} RQ=MEC/PNEC \end{array}$$

In Equation (1), RQ < 0.1 means low risk, < RQ < 1 means medium risk and, RQ > 1 means high ecological risk (219, 220).

Also in this equation, *PNEC* equals the *LC*50/1000 where **LC**_50_ is the chemical concentration that results in death to 50% of the studied population.

Instead of the three levels of risks of antibiotics (as well as other pharmaceuticals) shown above, the following four levels of risks (ecotoxicity) have been proposed by FASS (221): RQ ≤ 0.1 for insignificant risks, 0.1 ≤ RQ ≤ 1 for low risks, 1 ≤ RQ ≤ 10 for moderate risks and, RQ > 10 for high risk.

Many research studies report high risk quotients of partially treated and untreated wastewater reuse associated with the presence of erythromycin (222–224), clarithromycin (222), and ciprofloxacin (225). The risk quotients in these instances were mostly >1, suggesting high ecological risk (219, 220). This also shows the potential environmental impact of antibiotic residues that may enter surface water from reclaimed water used for irrigation. Chlortetracycline and oxytetracycline, were reportedly the most prevalent antibiotics found in livestock wastewater treatment plants (226) with enhanced hazard to ecosystems in Korea. Following a study on long-term wastewater irrigation, Christou et al. (178) concluded that the estimated threshold of toxicity concern (TTC) and high hazard quotients (HQ) values of pharmaceuticals observed, revealed that wastewater irrigation has an impact on human health, because the pharmaceutical gets internalized and bioaccumulated in tissues causing toxicity.

## Mitigating the Risks Associated with Wastewater Reuse

There are several approaches to consider mitigating the risks associated with the reuse of wastewater. These approaches have been captured in the World Health Organization (WHO) wastewater reuse guidelines (34, 151) and in sanitation safety plans (227). One approach that has proven to show positive results is the cessation of irrigation before harvesting (36, 228). The main risk reduction factor associated with cessation of irrigation is the decay of pathogens with time. Keraita et al. (228) studied the reduction of microbial contamination on wastewater-irrigated lettuce due to cessation of irrigation before harvesting and obtained a risk reduction of 0.65 log_10_ units for thermotolerant coliforms and 0.4 log_10_ helminth eggs per 100 g of lettuce. In addition, Sjölander (36) reported a decay rate of 0.4 day^−1^ for *Ascaris suum* on lettuce following cessation of irrigation before harvesting. The need to employ an optimum model to determine the appropriate time of die-off is thus imperative, particularly for interventions associated with health risk reduction (229). When accuracy in the actual prediction of the potential risk is involved, Oron et al. (230) recommended that research should incorporate definite acceptable risk criteria, more accurate dose-response modeling information including survival time of pathogen in treated wastewater. Information on uptake of pathogens by irrigated plants as well as the eating habits of the populations would provide a more pertinent impact (230). In relevance to these criteria such as plant uptake or eating habits of exposed population, and analytical tools' harmonization, Beaudequin et al. (231) proposed the use of a Bayesian network as a useful tool in situations where empirical data necessary for QMRA calculation requirements are insufficiently available. This may be integrated into other models to predict effectiveness of appropriate cessation period before harvest. The cessation may be used with other treatment technologies for wastewater before reuse (232).

In handling some viruses, Kobayashi et al. (232) have optimized a down-flow hanging sponge (DHS) reactor treating municipal wastewater. The optimization depicts that in DHS effluent for agricultural irrigation for 95% human population, a log_10_ reduction of 2.6 and 3.7 for norovirus GII in the DHS is attainable. This would, respectively, retain ≤ 10^−4^ and 10^−6^ DALYs loss per person per year (232). The impacts of using wastewater for irrigation on the one hand and post-harvest handling and storage contamination on the other, needs to be further addressed. The specific situation with the potential impact of internalization and uptake of pathogens as compared to deposition on outer surfaces need much more attention and documentation, before long-term handling and management practices can be issued and related to modes of application.

In addition, the specific situation with regards to chemical contaminants (e.g., uptake of antibiotics and other organic compounds) as well as the impact of use of these in livestock and among humans and the further fate in agricultural fields need to be addressed. Chen and Zhang (233) noted that constructed wetlands are effective for removing antibiotics and ARGs and compares favorably with mechanical wastewater treatment processes (*p* > 0.05). Modern processes involving ozonation, advanced oxidation, activated carbon, nano-filtration and reverse osmosis have also been recommended as treatment technologies (234).

Onsite filtration of the wastewater for irrigation during application may reduce the extent of farmland contamination. This may be considered in relation to riverbank filtration (RBF) proposed by Verbyla et al. (10). RBF may be accompanied with UV device for additional treatment before use (235). Toscano et al. (235) reported a significant (*p* < 0.05) average reduction of pathogen concentration of reclaimed water from 0.35 to 1.23 log units by using the UV system.

The guidelines by WHO on the reuse of wastewater in Agriculture (236) has provided a path to safeguard human health. If adequate care is taken, it is possible to achieve a better crop yield with wastewater effluents without constituting hazards (237, 238). Multiple barrier approach recommended in the guidelines is to protect the farmer and farm household with contacts to the wastewater being reused. It is also meant for protecting the food chain at critical control or entry points, especially in arid and semi-arid countries where reuse of treated or untreated wastewater is rampant (236). Sometimes, there can be the need for necessary steps and considerations of social marketing, incentive systems, awareness creation or education and local regulations (239). This would encompass a framework to protect both the farmers and the consumers.

## Conclusion

The reuse of partially treated or untreated wastewater for agricultural irrigation is widespread due to its availability and high nutrient content. The agricultural reuse of wastewater contributes to poverty alleviation especially in resource-constraint regions. Despite the many benefits associated with the practice serious health concerns still exist as presented in this review. The health impact is dependent on the concentration of the contaminants which varies, as shown in Table 1. The concentration of the contaminants is dependent on the type of wastewater, either treated, partially treated or untreated. The WHO wastewater reuse guidelines as well as the Sanitation Safety plans has been shown to protect public health, however the evidence presented in this review indicates a shortcoming in the implementation. This calls for a more concerted effort in adapting these guidelines/plans into local frameworks. The planning and implementation of these local frameworks should involve all stakeholders, such as policy makers, farmers, health professionals and the general public.

## Author Contributions

All authors listed have made a substantial, direct and intellectual contribution to the work, and approved it for publication.

### Conflict of Interest Statement

The authors declare that the research was conducted in the absence of any commercial or financial relationships that could be construed as a potential conflict of interest.
